# Epidemiology of Fall Injury in Rural Bangladesh

**DOI:** 10.3390/ijerph14080900

**Published:** 2017-08-10

**Authors:** Shirin Wadhwaniya, Olakunle Alonge, Md. Kamran Ul Baset, Salim Chowdhury, Al-Amin Bhuiyan, Adnan A. Hyder

**Affiliations:** 1 Johns Hopkins International Injury Research Unit, Department of International Health, Johns Hopkins Bloomberg School of Public Health, 615 N. Wolfe Street, Baltimore, MD 21205, USA; oalonge1@jhu.edu (O.A.); ahyder1@jhu.edu (A.A.H); 2 Center for Injury Prevention and Research, Bangladesh (CIPRB), House B162, Road 23, New DOHS, Mohakhali, Dhaka 1206, Bangladesh; kamran_baset@yahoo.co.uk (M.K.U.B.); smchow_dhaka@yahoo.com (S.C.); al-amin@ciprb.org (A.-A.B.)

**Keywords:** injury, fall injury, Bangladesh, LMICs

## Abstract

Globally, falls are the second leading cause of unintentional injury deaths, with 80% occurring in low-and middle-income countries. The overall objective of this study is to describe the burden and risk factors of falls in rural Bangladesh. In 2013, a large household survey covering a population of 1,169,593 was conducted in seven rural sub-districts of Bangladesh to assess the burden of all injuries, including falls. The recall periods for non-fatal and fatal injuries were six and 12 months, respectively. Descriptive, bivariate and multiple logistic regression analyses were conducted. The rates of non-fatal and fatal falls were 36.3 per 1000 and 5 per 100,000 population, respectively. The rates of both fatal and non-fatal falls were highest among the elderly. The risk of non-fatal falls was higher at extremes of age. Lower limb and waist injuries were frequent following a fall. Head injuries were frequent among infants (35%), while lower limb and waist injuries were frequent among the elderly (>65 years old). Injuries to all body parts (except the waist) were most frequent among men. More than half of all non-fatal falls occurred in a home environment. The injury patterns and risk factors of non-fatal falls differ by sociodemographic factors.

## 1. Introduction

Annually, about 4.7 million deaths are due to injury, and these account for about 8.5% of the global disease burden [1]. The majority (80%) of these deaths occur in low-and middle-income countries (LMICs) and the World Health Organization’s South-East Asia region has the highest unintentional injury mortality rate (80 per 100,000) and the highest rate for disability-adjusted life years (DALYs) due to unintentional injuries (3065 per 100,000) in the world [2,3]. Falls are the second leading cause of unintentional injury deaths, and the 13th leading cause of global years lived with disability (YLD) [1,2,3,4]. Between 2005–2015, global deaths due to fall increased by about 21%, and a major contributor for this was population growth and aging [1]. If left unaddressed, the burden of fall injuries is projected to increase by 100% by the year 2030 [5].

Fall can occur on the same level as a result of slipping or tripping, or at a different level, such as a fall from a height [2,5]. Several sociodemographic, occupational, health or medical, and environmental factors have been identified as risk factors for falls [2,5]. Globally, the burden of fall injuries is higher among the elderly (>65 years), and this burden increases with age [5]. However, children are also vulnerable to falls [6,7]. There is limited epidemiological data on fall injuries in LMICs [5].

Bangladesh, a lower-middle income country in the South Asia, has a high burden of disability as a result of injury, including falls [8]. Previous studies from Bangladesh have reported a high burden of fall-related mortality, morbidity and disability among children [9,10,11]. The government of Bangladesh has recognized injury prevention as a national priority [8]. However, most fall injury-related studies from Bangladesh have focused on specific population or group. An in-depth population-based study on the epidemiology of falls can provide information on risk factors and would help in planning effective prevention strategies. The overall goals of this study are to describe the burden of fall injuries, identify risk factors and patterns of fall injuries, and make recommendations to reduce the burden of falls in rural Bangladesh.

## 2. Materials and Methods

As part of the Saving of Lives from Drowning (SoLiD) project, a large baseline survey was conducted from June–November 2013 in seven purposively selected sub-districts—Matlab North, Matlab South, Daudkandi, Chandpur Sadar, Raiganj, Sherpur Sadar and Manohardi—covering a population of approximately 1.16 million people in 51 unions [12,13]. The data collection method for this survey has been described earlier, and is summarized below [14]. 

All data collectors received training on research methods, data collection tools, and ethics. Prior to starting with data collection, mapping and household listing was conducted in all villages. During this exercise, each household was assigned a serial number based on a predetermined format. All households in the selected sub-district were visited i.e., a census survey was conducted. A written consent was obtained prior to starting data collection. Data was collected from either the head of the household or any adult member of the household aged 18 years and older. Household, sociodemographic and injury data were collected for all individuals in each household. The birth history of all ever-married women who were between 15–49 years of age was obtained. Data on all injury mortality and morbidity were collected. Injury mortality data was collected over a 12-month recall period, while injury morbidity data was collected over a six-month recall period. Only injuries for which care was sought from either a formal or informal provider, and/or there was loss of work/daily activities for a day, were included in the survey.

Household, sociodemographic data and birth history were collected by one set of data collectors, who were also responsible for completing an injury notification form for any injury mortality and morbidity event that occurred during the recall period. Based on the outcomes recorded on the injury notification form, a second set of data collectors visited households to complete injury mortality/morbidity and mechanism forms. All of the forms were developed in English, translated to Bangla, and then back-translated and pilot-tested prior to data collection. 

A dichotomous variable for fall injury—yes or no—was created. In case of multiple falls, only one event per person was taken into consideration for analysis. Sub-district, age group, sex, education, marital status and occupation were categorical variables. Using principle component analysis, households were categorized into five quintiles: lowest, low, middle, high, and highest. Rates for fall-related mortality and morbidity by each category were calculated. These are expressed per 100,000 and per 1000 population for mortality and morbidity, respectively. 

Simple descriptive analysis and bivariate cross-tabulations were conducted [15]. To explore differences in fall-related mortality and morbidity by sociodemographic categories, the Chi-square test or Fisher’s exact test (when conditions for Chi-square were violated) were conducted [15]. For both fall mortality and morbidity, bivariate logistic regression analyses were used to study the association of each covariate with fall mortality and morbidity [15]. These covariates included sub-district, age group, sex, education, marital status, occupation, and socioeconomic status. Multivariate logistic regressions (MLR) were performed for fall morbidity using the same explanatory variables [15]. For non-fatal falls, a comparison of results from bivariate and MLR indicated an interaction between age and sex; hence, another model with an interaction term including age group and sex was run. The multivariate models without and with the interaction term were compared using the likelihood ratio test and the model with the interaction term was significant (<0.001). For fatal falls; age, education, marital status and occupation groups were re-categorized, as there were no fall-related deaths in some groups. To address the separation issue, Firth logit regression was run for fatal falls. In the Firth logit regression model; sub-district, age group, sex, education, marital status, occupation, and socioeconomic status were included as covariates. Injured body parts were combined into seven body regions: head and neck, face, chest, abdomen, upper limb, lower limb, and waist. The proportion of fall cases that had injury to a particular body region by age group and sex were calculated.

All analyses were carried out using STATA version 12 and 13 (StataCorp LP, College Station, Texas, USA), and *p* < 0.05 was considered statistically significant [16]. The Institutional Review Board of the Johns Hopkins Bloomberg School of Public Health (JHSPH), and the Ethics Review Committees of the International Center for Diarrhoeal Disease Research, Bangladesh (icddr, b), and Center for Injury Prevention and Research, Bangladesh (CIPRB), approved this study (JHSPH IRB 00004746).

## 3. Results

The baseline survey covered a total of 1,169,593 individuals in seven rural sub-districts of Bangladesh. About 39% of the population was children (<18 years), and about 55% were in the productive age group (18–64 years). The overall male to female ratio was 1:1.06. Nearly a quarter of the population did not have any formal education, and nearly half were married. About 35% were either retired, unemployed, or housewives. The distribution of the population in five wealth quintiles was nearly equal (Table 1).

### 3.1. Fatal Fall Injury 

A total 59 fatal falls were recorded in the survey, and the mortality rate in this population was 5 per 100,000 persons (Table 1). Fall mortality rates were highest in the Daudkandi sub-district (10.6 per 100,000 population). Nearly 66% of fatal falls were among the elderly (those above the age of 64 years), with a mortality rate of 54.6 per 100,000 population. Fall mortality rates were also higher among those with no formal education, and among the widowed (Table 1).

### 3.2 Non-Fatal Fall Injury 

Of the total 1,169,593 individuals who were surveyed, 42,259 reported to have experienced at least one fall injury in the six months preceding the survey. The incidence of non-fatal fall in this population was 36.3 per 1000 population. Fall morbidity rates were highest in the Daudkandi sub-district (67.5 per 1000 population), and among those above the age of 64 years (61 per 1000 population). Nearly 57% of falls were among women. Rates of non-fatal falls were also higher among those with no formal education, the widowed, the retired or unemployed, and housewives (Table 1).

**Figure 1:**
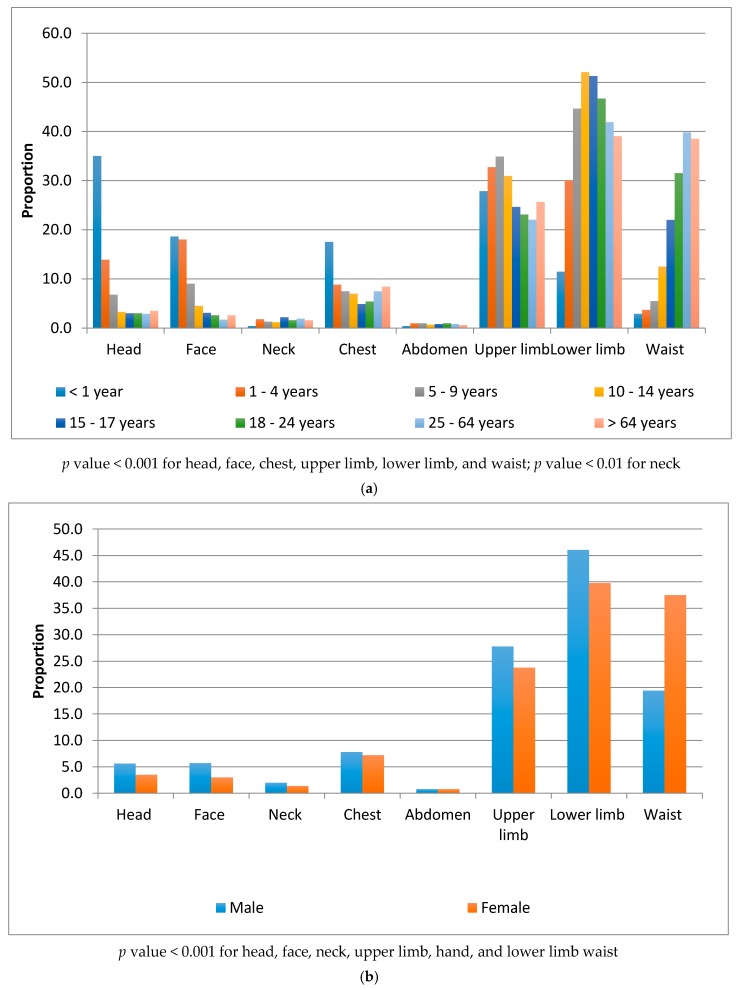
Body parts injured in non-fatal falls by age groups and sex. (**a**) Injured body parts by age groups; (**b**) Injured body parts by sex.

Lower limb (42.5%), upper limb (25.5%), and waist (29.6%) were the most common body regions injured in a fall. A statistically significant association was found between injured body parts and age and sex. Head, face and chest injuries were frequent in younger children (<4 years); upper limb injuries were frequent in older children (5–9 years old); lower limb injuries were frequent in young adults (10–24 years old); and waist injuries were frequent in adults over 24 years of age (Figure 1a). Men more frequently injured all the listed body regions, with the exception of the waist (*p* < 0.001, Figure 1b).

**Figure 2:**
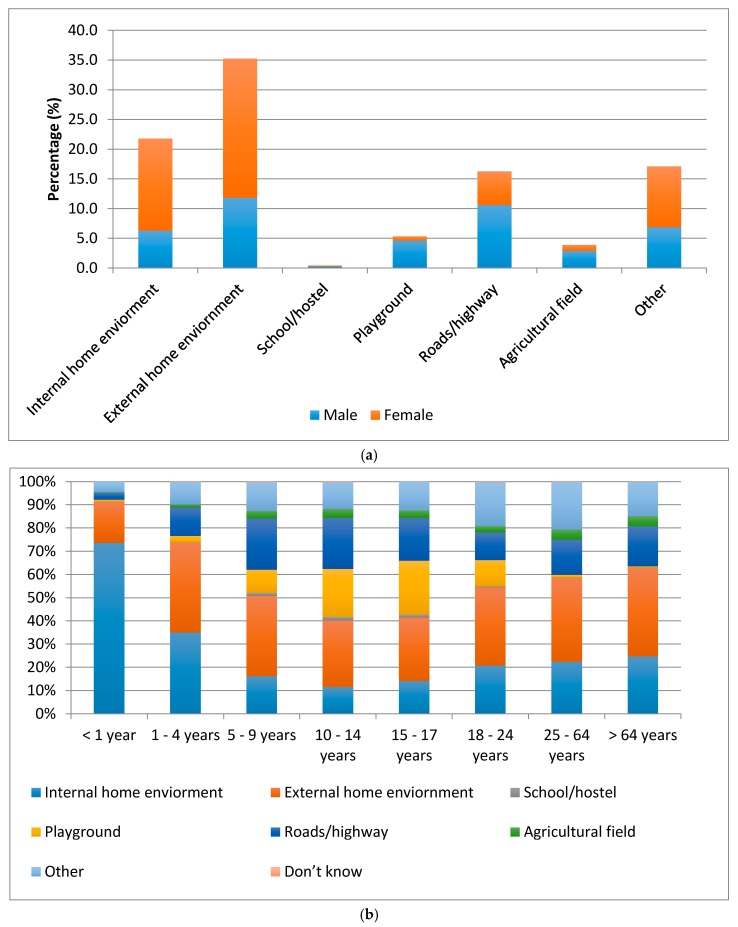
Place of non-fatal fall injuries by sex and age. (**a**) Place of injury by sex; (**b**) Place of injury by age group.

The majority (70%) of falls were at the same level, and most falls occurred in either external or internal home environments. Most falls among women were within a home environment, while the majority of falls among men were outside the home (Figure 2a). The majority of falls among children <4 years and adults (>18 years) were also in either internal or external home environments, while most falls among adolescents were outside the home (Figure 2b).

Nearly 70% of all falls had occurred at the same level as a result of slipping/tripping (66%) or stumbling (26%). Also, most of the same-level falls occurred on a sidewalk or street (62%), followed by within the home environment (18%), while most of the different-level falls were from a tree (27%), stairs (25%), or furniture (18%).

### 3.3. Factors Associated with Fatal Falls

Compared to children (<18 years), the elderly (>64 years) had higher odds of experiencing fatal falls. Education played a protective role for fall injury; those with any education were 70% less likely to sustain fatal falls compared with those with no formal education (Table 2). Individuals who were divorced, widowed, or separated had nearly six times higher odds of suffering fatal falls compared with those who were married. No statistically significant association was found between fatal falls and sub-district. No significant difference was found between fatal falls and socioeconomic status (Table 2). 

After adjusting for other covariates, no significant relationship was found between fatal falls and sub-district, age group, sex, marital status, occupation, or socioeconomic status (Table 2). Those with primary education were 60% less likely to experience fatal falls compared with those with no formal education.

### 3.4. Factors Associated with Non-Fatal Falls

Compared to Matlab North, the populations in Daudkandi and Matlab South had higher odds of sustaining non-fatal falls. The odds of experiencing non-fatal falls show a bimodal distribution by age group. After infancy, the odds increase for 1–4-year-olds, it then plateaus between the ages of 10 and 24 years, and then increases again among those above 24 years of age (Table 3). Women were 1.2 times likely to sustain non-fatal falls, compared with men. The odds of experiencing fall injury were highest among those with no formal education, and the odds reduced with increasing education level (*p* < 0.001, Table 3).

The widowed had 1.8 times higher odds of having non-fatal falls (*p* < 0.001) compared with married individuals. Also, compared with those involved in agriculture, the odds of experiencing falls were 1.6 times higher among the retired, the unemployed, and housewives (Table 3). Compared with those in the lowest socioeconomic status (SES) group, those in the highest SES group had a 10% lower risk of sustaining falls (*p* < 0.001). The relationship between non-fatal falls and the middle SES group was statistically significant, but not meaningful (Table 3).

After controlling for age, sex, education, marital status, occupation, socioeconomic status, and introducing the age–sex interaction terms; the odds of sustaining non-fatal falls for different sub-districts remained significant. The odds of fall injury were 2.9 times higher among 1–4-year-olds compared with infants. After adjusting for other covariates, the relationship between sex and non-fatal falls did not remain significant. However, even after adjusting, those widowed continued to have higher odds of sustaining falls compared with their reference group (Table 3). The odds of having fall injuries were also significantly higher among the unskilled and domestic laborers, students, and children, compared with those involved in agricultural work (Table 3). 

Female infants were 90% less likely to sustain falls compared with male infants (*p* < 0.001, Table 4). Among 1–4-year-olds, females were 2.7 times more likely to experience falls compared with 1–4-year-old males (*p* < 0.001, Table 4). However, 18–64-year-old females were 30% less likely to sustain falls compared with men in the same age category (Table 4). 

## 4. Discussion

To our knowledge, this is the first study describing the epidemiology of fall injuries among the general population in Bangladesh. Previous studies from Bangladesh have either focused on specific segments of the population or groups [9,10,17,18]. The rates of fatal and non-fatal falls were 5 per 100,000 population, and 36.3 per 1000 population, respectively. The rates of fatal and non-fatal falls were highest among the elderly. Other groups vulnerable to non-fatal falls included children, the widowed, unskilled/domestic laborers, and students. Residents of Matlab South and Daudkandi were also vulnerable for falls, while those in the highest SES group had a lower risk.

In our study, about 66% of all fatal falls were among the elderly. Falling can lead to physical disability, but it may also affect the mental, social and emotional well-being of the elderly [5]. In the last decade, Bangladesh has made significant achievements to improve health indicators, and the life expectancy at birth has also increased from 67.5 in 2004 to 71.6 years in 2014 [19]. With the increase in the aging population, the burden of fall injuries is also expected to increase in LMICs, including Bangladesh, and there is a need to focus on interventions that prevent falls among this age group [5,20].

In high-income settings, primary prevention interventions that promote physical activity and healthy lifestyles among the elderly have been implemented to prevent fall injuries [5,21]. Interventions for fall risk assessment and management are also found to be effective [21]. These programs may focus on the management of medications and health problems related to vision, foot, orthostatic hypotension, cardiovascular problems, gait, and balance [5]. Environmental modifications at home and within communities are also recommended [5]. Secondary prevention strategies such as the use of hip protectors to prevent hip fracture in the event of a fall have also been implemented [2,5]. Tertiary prevention strategies may focus on rehabilitation and improvement of functions to prevent disability following a fall. [5] However, there is a dearth of effective fall-prevention interventions from LMICs, including Bangladesh, and there is limited evidence on the acceptability of these interventions in this setting [20,22,23].

Children emerged as the other vulnerable group for fall injuries. Earlier studies from LMICs and Bangladesh have also highlighted this [6,7,10,17,18,24,25]. A multisite unintentional injury surveillance study conducted in five LMICs found that about half of all emergency department visits among children aged 0–12 years were as a result of falls [7]. Again, most fall injuries are found to occur in or around the home environment, and during play [6,7,10,17,18]. This could be because children in LMICs lack designated and safe play areas [26]. Strategies such as installing window guards, making environmental modifications in and around homes, and enforcing standards in playgrounds may also help prevent fall injuries among children [4].

Globally, elderly women are more likely to suffer from fall injuries compared with men [4]. However, no gender difference in the distribution of fatal and non-fatal falls among the elderly was found in this study. In our study, men in the productive age group (18–64 years) had a higher risk of falling compared with women in the same age category. In rural Bangladesh, men play an active role outside the home, and may be involved in high-risk behaviors and occupations that increase their risk of falling [2]. Previous studies have found falls to be more frequent among boys [6,7,10,17,18]. This could be because boys tend to play outside the house, and are exposed to greater risk compared with girls, who are mostly involved in household activities [17,18]. Contrary to earlier findings, in our study, 1–4-year-old girls had a relatively higher risk of sustaining non-fatal falls compared with boys in the same age group. Further research is required to explain the gender difference in this age group. 

In this study, falls among young children, women, and the elderly were more frequent within the home environment, while those among men and adolescents were more frequently outside. In Bangladesh, children, women, and the elderly tend to spend more time at home, and this can explain the higher incidence of falls within the home environment for these groups. In contrast, men and adolescents spend more time outside the home, and this can explain the higher incidence of falls outside the home environment [27].

Our study found a higher risk of fall injuries among the widowed. This could be because these individuals are more likely to live alone, lack social support, have lower income, and experience physical and mental health problems that increase their risk of falls [5]. In our study, we found that compared to those in the lowest SES group, those in the highest SES category had a slightly lower risk of falls. This may be due to differences in the designs and materials used for constructing houses amongst SES groups; the housing structure of the highest SES group may be safer and less injury-prone compared with those in the lowest SES category. 

In our study, the pattern and the distribution of injuries resulting from falls varied by age and sex. Young children were more likely to suffer from head and chest injuries; older children and adolescents were more likely to suffer from limb injuries; while young adults and the elderly were more likely to suffer from lower limb and waist injuries. These findings are comparable with those found in other studies [27,28]. 

The risk of falls also differed by sub-districts. Residents of Matlab South and Daudkandi were found to be more vulnerable to falls. This may be related to environmental factors. However, these were not captured in this survey, and this could be explored in future studies.

Results from the study may help in planning injury-prevention interventions and policies in Bangladesh. However, the findings of this study cannot be generalized to other areas in Bangladesh, as this study was conducted exclusively in rural Bangladesh. Previous studies have shown that rural areas have a higher burden of falls compared with urban areas [17,29]. Another limitation of the study could be the recall bias. In this study, the recall period for fatal and non-fatal fall was six and 12 months, respectively. Respondents are more likely to recall recent, severe, or fatal falls, and there may be underreporting. Other factors such as medication and substance use, or medical conditions such as poor vision, vertigo, stroke and musculoskeletal diseases, all may impair judgment, muscle strength, coordination or balance, and result in fall injuries [5]. Environmental factors such as poor infrastructure, building design, or lighting may also increase the risk of falling, especially for children who are inexperienced, and the elderly [5]. In this survey, data on these associated factors were not collected. While this study attempts to explore associations between fatal falls and sociodemographic factors, there may be some analytic limitations, as only 59 fatal falls were recorded in this survey.

## 5. Conclusions

Bangladesh has a high burden of fall-related mortality and morbidity. Populations at age extremes, and men in the productive age group, were found to be most vulnerable for falls. With increasing life expectancy, the burden of falls among the older population is expected to increase. Other groups that were vulnerable for falls included the widowed, those with lower education, unskilled/domestic laborers, students, and those in the lowest SES group. Interventions targeting these specific groups may help reduce the burden of falls in Bangladesh. Since most falls among children and elderly occur in the home environment, modifications in and around homes could be a potential strategy. Other strategies could be fall risk assessment and management. However, there is a dearth of evidence on the acceptability and effectiveness of these strategies in LMICs; as a result, future research on fall prevention interventions is suggested.

## Figures and Tables

**Table 1:** Sociodemographic characteristics of fatal and non-fatal fall injury patients in rural Bangladesh.

	Fatal Fall Injury (*N* = 59)		*p* Value	Non-Fatal Fall Injury (*N* = 42,259)		*p* Value	Total (*N* = 1,169,593)
	*N*	Rate Per 100,000 Population (95% CI)		*N*	Rate Per 1000 Population (95% CI)		*N* (%)
**Sub-district**							
Matlab North	21	7.9 (4.5–11.3)	0.030 *	12,058	45.6 (44.8–46.4)	<0.001 ***	265,897 (22.7)
Matlab South	15	7.2 (3.5–10.8)		11,435	54.8 (53.8–55.8)		209,772 (17.9)
Chandpur Sadar	3	2.3 (0.0–5.0)		4570	35.8 (34.8–36.8)		128,356 (11.0)
Raiganj	2	1.9 (0.0–4.6)		3554	34.3 (33.1–35.4)		104,357 (8.9)
Sherpur Sadar	8	3.5 (1.1–5.9)		4059	17.9 (17.3–18.4)		228,519 (19.5)
Manohardi	7	3.4 (0.9–6.0)		4680	23.0 (22.4–23.7)		204,319 (17.5)
Daudkandi	3	10.6 (0.0–22.5)		1903	67.5 (64.6–70.5)		28,373 (2.4)
**Age group**							
<1 year	0	-	-	280	13.0 (11.5–14.5)	<0.001 ***	22,141 (1.9)
1–4 years	0	-		2927	32.5 (31.3–33.6)		90,523 (7.7)
5–9 years	3	2.2 (0.0–4.6)		4453	31.9 (31.0–32.8)		139,728 (12.0)
10–14 years	1	0.7 (0.0–2.1)		3848	27.1 (26.2–27.9)		142,121 (12.2)
15–17 years	0	-		1435	23.1 (21.9–24.3)		62,098 (5.3)
18–24 years	2	1.5 (0.0–3.6)		2879	21.6 (20.8–22.4)		133,534 (11.4)
25–64 years	14	2.8 (1.3–4.2)		22,273	44.0 (43.4–44.6)		508,059 (43.4)
>64 years	39	54.6 (37.5–71.8)		4164	61.0 (59.2–62.8)		71,389 (6.1)
**Sex**							
Male	31	5.5 (3.5–7.4)	0.538	18,312	32.4 (32.0–32.9)	<0.001 ***	567,674 (48.5)
Female	28	4.7 (2.9–6.4)		23,947	40.0 (39.5–40.5)		601,919 (51.5)
**Education**							
No education	39	13.2 (9.1–17.4)	<0.001 ***	12,608	43.2 (42.5–43.9)	<0.001 ***	295,314 (25.3)
Primary	10	2.5 (0.9–4.0)		15,734	38.7 (38.1–39.2)		407,923 (34.9)
Secondary	8	2.8 (0.8–4.7)		9228	31.9 (31.3–32.6)		289,658 (24.8)
A levels	1	2.2 (0.0–6.5)		1079	23.7 (22.3–25.1)		45,618 (3.9)
College	0	-		299	22.2 (19.7–24.7)		13,526 (1.2)
Advanced/Professional degree	0	-		100	21.3 (17.2–25.4)		4729 (0.4)
Not applicable (under 5 children)	0	-		3207	28.7 (27.7–29.7)		112,664 (9.6)
**Marital status**							
Married	33	5.8 (3.8–7.8)	<0.001 ***	23,845	42.0 (41.5–42.5)	<0.001 ***	571,206 (48.8)
Never married	2	0.9 (0.0–2.1)		5052	22.2 (21.6–22.9)		227,319 (19.4)
Divorced	0	-		116	36.3 (29.8–42.8)		3220 (0.3)
Widowed	20	37.7 (21.2–54.2)		3695	71.6 (69.3–73.8)		53,096 (4.5)
Separated	0	-		128	47.4 (39.4–55.4)		2717 (0.2)
Not applicable	4	1.3 (0.0–2.5)		9423	30.3 (29.7–30.9)		312,035 (26.7)
**Occupation**							
Agriculture	9	8.6 (3.0–14.2)	-	3440	33.1 (32.1–34.2)	<0.001 ***	104,956 (9.0)
Business	3	4.9 (0.0–10.4)		1518	24.8 (23.5–26.0)		61,661 (5.3)
Skilled labor (Professional)	5	5.6 (0.7–10.5)		2250	25.3 (24.3–26.4)		89,151 (7.6)
Unskilled/domestic labor	0	-		894	36.6 (34.3–39.0)		24,520 (2.1)
Rickshaw/bus (transport worker)	1	5.9 (0.0–17.4)		445	26.2 (23.8–28.6)		17,037 (1.5)
Students	3	1.0 (0.0–2.1)		8580	27.5 (26.9–28.0)		312,537 (26.7)
Retired/unemployed/housewife	36	8.8 (5.9–11.7)		20,716	51.1 (50.4–51.7)		408,583 (34.9)
Not applicable (children)	0	-		4195	29.3 (28.4–30.1)		144,454 (12.4)
Not applicable (others)	2	33.6 (0.0–80.2)		195	33.7 (29.1–38.4)		5948 (0.5)
**Socioeconomic status**							
Lowest	7	3.3 (0.9–5.8)	0.653	7714	36.7 (35.9–37.5)	<0.001 ***	211,601 (18.1)
Low	11	5.0 (2.1–8.0)		7927	36.4 (35.6–37.2)		218,695 (18.7)
Middle	11	4.6 (1.9–7.3)		9051	38.2 (37.4–39.0)		238,371 (20.4)
High	16	6.5 (3.3–9.6)		9051	36.7 (36.0–37.4)		247,716 (21.2)
Highest	14	5.5 (2.6–8.4)		8516	33.8 (33.1–34.5)		253,201 (21.7)

Missing: education for 0.01% (n = 151), occupation for 0.06% (n = 737); *** *p* < 0.001, * *p* < 0.05.

**Table 2:** Factors associated with fatal fall injuries in Bangladesh.

	Unadjusted		Adjusted ^	
	Odds Ratio (95% CI)	*p* Value	Odds Ratio (95% CI)	*p* Value
**Sub-district**				
Matlab North	1	-	1	-
Matlab South	0.9 (0.5–1.8)	0.769	1.0 (0.5–1.9)	0.976
Chandpur Sadar	0.3 (0.1–1.0)	0.049 *	0.4 (0.1–1.2)	0.111
Raiganj	0.2 (0.1–1.0)	0.056	0.3 (0.1–1.2)	0.098
Sherpur Sadar	0.4 (0.2–1.0)	0.050	0.6 (0.3–1.4)	0.229
Manohardi	0.4 (0.2–1.0)	0.056	0.5 (0.2–1.2)	0.123
Daudkandi	1.3 (0.4–4.5)	0.636	1.4 (0.4–4.3)	0.567
**Age group**				
<18 years	1	-	1	-
18–24 years	1.7 (0.3–9.3)	0.536	5.0 (0.1–333.1)	0.456
25–64 years	3.1 (1.0–9.6)	0.043 *	5.9 (0.1–500.6)	0.431
>64 years	62.4 (22.3–174.6)	<0.001 ***	71.7 (0.8–6285.6)	0.061
**Sex**				
Male	1	-	1	-
Female	0.9 (0.5–1.4)	0.538	0.5 (0.3–1.1)	0.107
**Education**				
No education (includes under 5 children)	1	-	1	-
Primary	0.3 (0.1–0.5)	<0.001 ***	0.4 (0.2–0.9)	0.023 *
Secondary and higher	0.3 (0.1–0.5)	<0.001 ***	0.5 (0.2–1.1)	0.099
**Marital status**				
Married	1	-	1	-
Never married	0.2 (0.0–0.6)	0.010 **	1.5 (0.3–8.3)	0.655
Divorced/widowed/separated	5.9 (3.4–10.2)	<0.001 ***	2.1 (1.0–4.3)	0.053
Not applicable	0.2 (0.1–0.6)	0.004 **	11.1 (0.1–996.8)	0.294
**Occupation**				
Agriculture	1	-	1	-
Business	0.6 (0.2–2.1)	0.395	1.2 (0.3–4.2)	0.784
Skilled labor (Professional)	0.7 (0.2–2.0)	0.447	1.8 (0.6–5.7)	0.291
Unskilled/semi-skilled labor	0.3 (0.0–2.2)	0.228	0.8 (0.1–4.5)	0.779
Students	0.1 (0.0–0.4)	0.001 **	0.5 (0.0–5.7)	0.598
Retired/unemployed/housewife	1.0 (0.5–2.1)	0.942	1.1 (0.4–2.7)	0.843
Not applicable	0.2 (0.0–0.7)	0.017 *	0.1 (0.0–1.9)	0.138
**Socioeconomic status**				
Lowest	1		1	
Low	1.5 (0.6–3.9)	0.386	1.6 (0.6–4.2)	0.303
Middle	1.4 (0.5–3.6)	0.491	1.5 (0.6–3.9)	0.362
High	2.0 (0.8–4.7)	0.140	2.1 (0.9–5.2)	0.094
Highest	1.7 (0.7–4.1)	0.267	1.8 (0.7–4.6)	0.203

*** *p* < 0.001, ** *p* < 0.01, * *p* < 0.05, ^ firthlogit regression.

**Table 3:** Factors associated with non-fatal fall injuries in Bangladesh.

	Unadjusted		Adjusted #	
	Odds Ratio (95% CI)	*p* Value	Odds Ratio (95% CI)	*p* Value
**Sub-district**				
Matlab North	1	-	1	-
Matlab South	1.2 (1.2–1.2)	<0.001 ***	1.2 (1.2–1.3)	<0.001 ***
Chandpur Sadar	0.8 (0.8–0.8)	<0.001 ***	0.8 (0.8–0.8)	<0.001 ***
Raiganj	0.7 (0.7–0.8)	<0.001 ***	0.7 (0.7–0.8)	<0.001 ***
Sherpur Sadar	0.4 (0.4–0.4)	<0.001 ***	0.4 (0.4–0.4)	<0.001***
Manohardi	0.5 (0.5–0.5)	<0.001 ***	0.5 (0.5–0.5)	<0.001 ***
Daudkandi	1.5 (1.4–1.6)	<0.001 ***	1.5 (1.4–1.6)	<0.001 ***
**Age group**				
<1 year	1	-	1	-
1–4 years	2.5 (2.3–2.9)	<0.001 ***	2.9 (2.4–3.4)	<0.001 ***
5–9 years	2.5 (2.2–2.8)	<0.001 ***	1.1 (0.9–1.2)	0.349
10–14 years	2.1 (1.9–2.4)	<0.001 ***	1.0 (0.9–1.1)	0.640
15–17 years	1.8 (1.6–2.0)	<0.001 ***	0.9 (0.8–1.0)	0.012 *
18–24 years	1.7 (1.5–1.9)	<0.001 ***	0.7 (0.6–0.8)	<0.001 ***
25–64 years	3.5 (3.1–3.9)	<0.001 ***	0.8 (0.7–0.8)	<0.001 ***
> 64 years	4.9 (4.4–5.6)	<0.001 ***	-	-
**Sex**				
Male	1	-	1	-
Female	1.2 (1.2–1.3)	<0.001 ***	0.9 (0.7–1.2)	0.550
**Education**				
No education	1	-	1	-
Primary	0.9 (0.9–0.9)	<0.001 ***	1.1 (1.0–1.1)	<0.001 ***
Secondary	0.7 (0.7–0.8)	<0.001 ***	0.9 (0.9–0.9)	<0.001 ***
A levels	0.5 (0.5–0.6)	<0.001 ***	0.8 (0.7–0.8)	<0.001 ***
College	0.5 (0.4–0.6)	<0.001 ***	0.7 (0.6–0.8)	<0.001 ***
Advanced/Professional degree	0.5 (0.4–0.6)	<0.001 ***	0.6 (0.5–0.8)	<0.001 ***
Not applicable (under 5 children)	0.7 (0.6–0.7)	<0.001 ***	0.3 (0.3–0.4)	<0.001 ***
**Marital status**				
Married	1	-	1	-
Never married	0.5 (0.5–0.5)	<0.001 ***	0.6 (0.6–0.7)	<0.001 ***
Divorced	0.9 (0.7–1.0)	0.108	0.9 (0.7–1.0)	0.115
Widowed	1.8 (1.7–1.8)	<0.001 ***	1.2 (1.2–1.3)	<0.001 ***
Separated	1.1 (0.9–1.4)	0.165	1.1 (0.9–1.3)	0.302
Not applicable	0.7 (0.7–0.7)	<0.001 ***	0.7 (0.6–0.7)	<0.001 ***
**Occupation**				
Agriculture	1	-	1	-
Business	0.7 (0.7–0.8)	<0.001 ***	0.7 (0.7–0.8)	<0.001 ***
Skilled labor (Professional)	0.8 (0.7–0.8)	<0.001 ***	0.8 (0.8–0.8)	<0.001 ***
Unskilled/domestic labor	1.1 (1.0–1.2)	0.006 **	1.1 (1.0–1.2)	0.029 *
Rickshaw/bus (transport worker)	0.8 (0.7–0.9)	<0.001 ***	0.8 (0.7–0.9)	<0.001 ***
Students	0.8 (0.8–0.9)	<0.001 ***	1.2 (1.1–1.3)	<0.001 ***
Retired/unemployed/housewife	1.6 (1.5–1.6)	<0.001 ***	1.1 (1.0–1.1)	0.079
Not applicable (children)	0.9 (0.8–0.9)	<0.001 ***	1.2 (1.1–1.3)	0.001 **
Not applicable (others)	1.0 (0.9–0.2)	0.807	1.3 (1.1–1.5)	0.002 **
**Socioeconomic status**				
Lowest	1	-	1	-
Low	1.0 (1.0–1.0)	0.650	1.0 (0.9–1.0)	0.145
Middle	1.0 (1.0–1.1)	0.009 **	1.0 (0.9–1.0)	0.043 *
High	1.0 (1.0–1.0)	0.942	1.0 (0.9–1.0)	0.002 *
Highest	0.9 (0.9–0.9)	<0.001 ***	0.9 (0.9–0.9)	<0.001 ***

# Adjusted for Age group and sex interaction; *** *p* < 0.001, ** *p* < 0.01, * *p* < 0.05.

**Table 4:** Adjusted odds of fall injuries by age and sex.

	Odds Ratio (95% CI)	*p*-Value
<1 year female compared to <1 year male	0.1 (0.0–0.1)	<0.001 ***
1–4 years female compared to 1–4 years male	2.7 (1.9–3.9)	<0.001 ***
5–9 years female compared to 5–9 years male	1.0 (0.8–1.3)	0.894
10–14 years female compared to 10–14 years male	1.0 (0.7–1.2)	0.710
15–17 years female compared to 15–17 years male	0.8 (0.6–1.1)	0.115
18–24 years female compared to 18–24 years male	0.7 (0.5–0.8)	0.001 **
25–64 years female compared to 25–64 years male	0.7 (0.6–0.9)	0.006 **
>65 years female compared to >65 years male	0.9 (0.7–1.2)	0.550

*** *p* < 0.001, ** *p* < 0.01.

## Abbreviations

aOR	Adjusted odds ratio
CIPRB	Center for Injury Prevention and Research, Bangladesh
CI	Confidence interval
icddr, b	International Center for Diarrhoeal Disease Research, Bangladesh
JHSPH	Johns Hopkins Bloomberg School of Public Health
LMIC	Low-and middle-income countries
MLR	Multivariate logistic regressions
OR	Odds ratio
SES	Socioeconomic status
YLD	Years lived with disability
